# The future of psychiatry and psychotherapy - An Early Career psychiatrist’s view

**DOI:** 10.1192/j.eurpsy.2024.1708

**Published:** 2024-08-27

**Authors:** F. H. Kraxner

**Affiliations:** Department of Psychiatry and Psychotherapy, Hospital of Affoltern am Albis, Affoltern am Albis, Switzerland

## Abstract

**Introduction:**

Psychiatry is one of the most fast developing and agile discipline within human medicine. But more work is necessary to complete these advances.

**Objectives:**

I address the following questions:

How does the future of psychiatry look in the eyes of early career psychiatrists?

What strengths, weaknesses opportunities and threats will come?

And what can we learn from different mental health systems and reagions?

**Methods:**

Oral or written statements to the raised questions followed optimally by a discussion

**Results:**

In low- and middle-income countries, a vast majority of people with mental disorders do not receive adequate treatment. Even in high income countries, roughly a third of people with severe forms of mental illness are not receiving the appropriate therapy. Laws concerning mental health are outdated in different countries. The protection of the human rights of the mentally ill is still incomplete and imperfect. The emphasis on economic gain and the digitalization of medicine in recent years has not helped. And new technical advancements such as artifical intelligence are becoming more important.

**Conclusions:**

More discussion needs to be done on the identity and understanding of the psychiatric profession.

**Disclosure of Interest:**

None Declared

